# A new trick for an old dog: The application of mifepristone in the treatment of adenomyosis

**DOI:** 10.1111/jcmm.14866

**Published:** 2019-12-08

**Authors:** Xuan Che, Jianzhang Wang, Jiayi He, Qin Yu, Wenting Sun, Shuyi Chen, Gen Zou, Tiantian Li, Xinyue Guo, Xinmei Zhang

**Affiliations:** ^1^ Women’s Hospital School of Medicine Zhejiang University Hangzhou China; ^2^ Jiaxing University Affiliated Women and Children Hospital Jiaxing China

**Keywords:** adenomyosis, apoptosis, invasion, mifepristone, migration, proliferation

## Abstract

Adenomyosis is also called internal endometriosis and affects about 20% of reproductive‐aged women. It seriously reduces life quality of patients because current drug therapies face with numerous challenges. Long‐term clinical application of mifepristone exhibits wonderful therapeutic effects with mild side‐effects in many disorders since 1982. Since adenomyosis is a refractory disease, we investigate whether mifepristone can be applied in the treatment of adenomyosis. In this study, we investigated the direct effects of mifepristone on human primary eutopic endometrial epithelial cells and stromal cells in adenomyosis. We found that mifepristone causes cell cycle arrest through inhibiting CDK1 and CDK2 expressions and induces cell apoptosis via the mitochondria‐dependent signalling pathway in endometrial epithelial cells and stromal cells of adenomyosis. Furthermore, mifepristone inhibits the migration of endometrial epithelial cells and stromal cells through decreasing CXCR4 expression and restricts the invasion of endometrial epithelial cells via suppression of epithelial‐mesenchymal transition in adenomyosis. We also found that mifepristone treatment decreases the uterine volume, CA125 concentration and increases the haemoglobin concentration in serum for adenomyosis patients. Therefore, we demonstrate that mifepristone could serve as a novel therapeutic drug in the treatment of adenomyosis, and therefore, the old dog can do a new trick.

## 1 INTRODUCTION

Adenomyosis is also called internal endometriosis and characterized by the invasion and migration of endometrial glands and stroma into the uterine myometrium. It is a common disease and affects about 20% of reproductive‐aged women. Although adenomyosis is a chronic benign disease, it causes severe symptoms to the patients such as enlarged uterus, hypermenorrhoea, severe dysmenorrhoea and infertility.^1^, ^2^ Furthermore, adenomyosis has some tumour‐like properties and malignant behaviours, such as aberrant proliferation, invasion and migration and therefore is a refractory and recurrent disease, which seriously reduces women's quality of life and leads to a heavy burden on the cost of health care.^3^, ^4^, ^5^, ^6^, ^7^

The treatment options for adenomyosis are summarized by Soave *et al* 8 and mainly include medical treatment, high intensity focused ultrasound (HIFU), surgery and combination therapy.^8^ Since hysterectomy is not accepted by most patients, drug therapy is still the first‐line treatment for adenomyosis.^9^ However, numerous challenges are emerging including notable side‐effects, limited efficacy and heavy financial burden. Therefore, the novel treatments are still deserved to be investigated. Mifepristone is the first and one of the most widely used selective progesterone receptor modulator since 1982.^10^ Until now, mifepristone has been proved to be effective in the treatment of endometriosis, uterine fibroids^11^, ^12^ and a variety of cancers including glioblastoma multiforme, lung cancer, cervical cancer, ovarian cancer and endometrial cancer.^13^, ^14^, ^15^, ^16^ Mifepristone exhibits wonderful clinical efficacies in more and more human diseases than what we originally thought following current understanding of mechanism of action of mifepristone. Long‐term clinical application of mifepristone proves that it is well tolerated and its side‐effects are generally mild. Besides, mifepristone has a very low price of long‐term treatment.^17^ Since this old dog, mifepristone, exhibits so many advantages and adenomyosis is a refractory disease, we want to know whether it can have a new trick in the treatment of adenomyosis.

Adenomyosis results from the increased invasive characteristics of the eutopic endometrial cells including increased proliferation, decreased apoptosis and angiogenesis, and these changes enhance the migratory capacity of eutopic endometrial cells into the myometrium and lead to the excessive proliferation of ectopic endometrial cells in the myometrium.^18^, ^19^ So eutopic endometrial cells, including endometrial epithelial cells and stromal cells, play crucial roles in the initiation and progression of adenomyosis.^20^ Obviously, drug therapy for adenomyosis should be based on the above‐mentioned pathogenesis of adenomyosis. In this study, we investigated whether the old and wonderful drug could be applied for the treatment of this painful and refractory disease. Our study is performed to provide the laboratory evidence and clinical basis for the treatment of adenomyosis with mifepristone.

## 2 MATERIALS AND METHODS

### 2.1 Patients and sample collection

This study was approved by Ethics Committee of Women's Hospital, School of Medicine, Zhejiang University and registered in Chinese Clinical Trial Registry (ChiCTR1800015514). Twenty adenomyosis patients were involved after informed written consent. No hormone or similar drugs were used for 6 months before treatment. The patients were treated with mifepristone by oral administration at 5 mg per day for 3 months. The diagnosis of adenomyosis was confirmed by imaging or histological examination. Adenomyosis endometrium and corresponding ectopic lesions in myometrium were collected during surgery. Samples were all collected in the proliferative phase of the menstrual cycle.

### 2.2 Isolation and identification of endometrial epithelial and stromal cells of adenomyosis

Endometrial tissues were obtained from hysterectomy samples of adenomyosis patients. The collected tissue was put into a culture dish and washed with FBS‐free medium. Tissues were minced into 1 × 1 × 1 mm^3^ pieces and digested with 1 mg/mL collagenase type III 37°C for 60 minutes, and then, the endometrial cells were separated by two sequential filtrations of 200‐ and 70‐μm cell strainer. Subsequently, endometrial epithelial cells were re‐suspended in primary epithelial growth medium (PriCells) and endometrial stromal cells were cultivated in Dulbecco's modified Eagle's medium (DMEM)/F12 medium (Thermo Fisher) supplemented with 10% FBS (Sigma‐Aldrich). The isolated endometrial epithelial cells and stromal cells were identified by immunofluorescent staining using epithelial marker pancytokeratin and mesenchymal marker vimentin as described in our previous study.^21^

### 2.3 The cell‐counting kit‐8 assay

The viability of endometrial epithelial and stromal cells with mifepristone treatment was detected by the cell‐counting kit‐8 (CCK‐8) assay (Biosharp). According to the manufacturer's protocol, the CCK‐8 reagent was added to each well and cells were incubated at 37°C for 2 hours. The absorbance at 450 nm (optical density) was measured and used to represent the cell viability. Each experiment was performed in three parallel wells and repeated for three times.

### 2.4 Cell migration assay

Cell migration ability was evaluated by transwell chamber assay using 24‐well plates with 8.0‐μm pore size membranes (BD Biosciences). To study the effect of mifepristone on the migratory ability of endometrial cells, endometrial epithelial and stromal cells were pre‐treated with mifepristone or AMD3100 (the CXCR4 inhibitor) in different concentrations (0, 50, 100 and 200 μmol/L for mifepristone and 0, 0.1, 1.0 and 10 μg/mL for AMD3100, respectively) in this experiment. The same number of endometrial cells were added into the upper chamber of the insert in 200 μL of serum‐free medium, while the lower chamber contained growth media with 10% FBS. After 24‐hour incubation, cells in the upper chamber were removed with a cotton swab and the migrated cells in the lower chamber were fixed with methanol, stained with crystal violet and counted with a microscope (Olympus). The number of cells that passed through the membrane was defined as migrated cell number.

### 2.5 High‐throughput sequencing

Primary endometrial epithelial cells were treated with mifepristone in three different concentrations (0, 50 and 100 μmol/L) for 24 hours in this experiment. The endometrial epithelial cells were from three biologically independent samples, and the data were shown in triplicate. Total RNAs were isolated using TRIzol reagent (Life Technologies) according to the handbook of the kit. The above RNAs were subjected to mRNA high‐throughput sequencing analysis by The Beijing Genomics Institute (BGI). The differential genes were clustered, and the heat map was plotted using the OmicShare tools.

### 2.6 Cell cycle analysis

Primary endometrial epithelial and stromal cells were treated with mifepristone in different concentrations (0, 50, 100 and 200 μmol/L) for 24 hours in this experiment. According to the protocol of Cell Cycle Staining Kit (LIANKE), about 1 × 10^6^ cells were harvested and washed with cold PBS for twice, and then were fixed by 75% ice ethanol for 24 hours. Cells were re‐suspended in PBS containing 40 μg/mL propidium iodide and 0.1 mg/mL RNase in dark room for 30 minutes at 37°C. Data analysis was performed using the BD CellQuest Pro software.

### 2.7 Flow cytometry assay

The percentage of cell apoptosis was assessed by flow cytometry assay according to the protocol of Annexin V‐FITC/PI Apoptosis Detection Kit (Beyotime) in this experiment. Briefly, cells were washed with cold PBS and re‐suspended in the binding buffer at a concentration 1 × 10^6^ cells/mL, and each tube of mixture was incubated with Annexin V‐FITC and propidium iodide for 15 minutes at room temperature. Data analysis was performed using the BD Cell Quest Pro software.

### 2.8 TUNEL

Apoptotic cells in the myometrium of adenomyosis uterus were further detected using a TUNEL colorimetric staining (In situ apoptosis detection kit, Invitrogen) according to the manufacturer's instructions. TUNEL staining was performed with fluorescein‐dUTP for apoptotic cell nuclei, and DAPI was used to stain all cell nuclei. After labelling, the TUNEL‐positive cells were visualized and counted under a fluorescence microscopy.

### 2.9 Quantitative real‐time PCR

The protocol of reverse transcription‐polymerase chain reaction (RT‐PCR) is based on protocol of Transcriptor First Strand cDNA Synthesis Kit (Takara). Total RNA (1 μg) was used as the template for the cDNA synthesis reaction with random primers. RT‐PCR was performed using SYBR Premix Ex Taq TM Kit (Takara) with ABI 7500 real‐time PCR system (Thermo, MMAS, USA). The nucleotide sequences of CDK1 were designed as follows: sense 5′‐AAGCCGGGATCTACCATACC‐3′ and antisense 5′‐TTTCATGGCTACCACTTGACC‐3′; the nucleotide sequences of CDK2 were as follows: sense 5′‐GACCAGCTCTTCCGGATCTT‐3′ and antisense 5′‐ACAAGCTCCGTCCATCTTCA‐3′; the nucleotide sequences of CXCR4 were as follows: sense 5′‐CTCCTCTTTGTCATCACGCTTCC‐3′ and antisense 5′‐GGATGAGGACACTGCTGTAGAG‐3′; the nucleotide sequences of N‐cadherin were as follows: sense 5′‐GATGTTGAGGTACAGAATCGT‐3′ and antisense 5′‐GGTCGGTATGGATGGCGA‐3′. As an internal control, GAPDH was also amplified and the nucleotide sequence for the primers was as follows: sense 5′‐GCCATCAATGACCCCTTCATT‐3′ and antisense 5′‐TGACGGTGCCATGGAATTT‐3.

### 2.10 Western blotting

The endometrial epithelial cells and stromal cells were treated with mifepristone at different concentrations (0, 50, 100 and 200 μmol/L, respectively) for 48 hours, and then, proteins were extracted as previously described.^22^ Equal amounts of total protein were electrophoresed in SDS‐PAGE gels and were transferred to PVDF membranes, and followed by overnight incubation with primary antibodies against cyclin E (Santa Cruz, 1:500), cyclin B (Santa Cruz, 1:500), CDK2 (Santa Cruz, 1:1000) and CDK1 (Abcam, 1:1000), CXCR4 (Abcam, 1:1000), N‐cadherin (Abcam, 1:1000), caspase‐3 and cleaved caspase‐3 (CST, 1:1000). Then PVDF membranes were incubated with secondary antibodies. Blots were detected using an enhanced chemiluminescence substrate kit (Thermo Fisher).

### 2.11 Immunohistochemical analysis

The sections were from adenomyosis samples, and immunohistochemical assay was performed as previously described.^23^ The results were evaluated by two independent investigators using a semi‐quantitative scale. A semi‐quantitative grading of CXCR4 expression was represented as the sum of percentage score and intensity score. Scores that correspond to the percentages of staining cells were defined as follows: 0 for no documented positive staining cell, 1 for the 25% positive staining cells, 2 for >25% and 50%, and 3 for >50%. Moreover, in terms of intensity of the stain, the following scores were designated as follows: 0 for no documented stains, 1 for a weak, 2 for moderate and 3 for high.^24^

### 2.12 Statistical tests

SPSS program version 19.0 and Graph Pad Prism 5 software were used for statistical analysis. Data are presented as the mean ± Standard Error of Mean (SEM). *P* values were determined by the two‐tailed Student's *t* test or Mann‐Whitney *U* test when comparing two groups and by a one‐way ANOVA when comparing more than two groups. Statistical difference was considered to be significant at a value of *P* < .05 (*), highly significant at a value of *P* < .01 (**) and extremely significant when *P* < .001 (***).

## 3 RESULTS

### 3.1 Mifepristone decreases the viability of endometrial epithelial cells and stromal cells in adenomyosis

**Figure 1:**
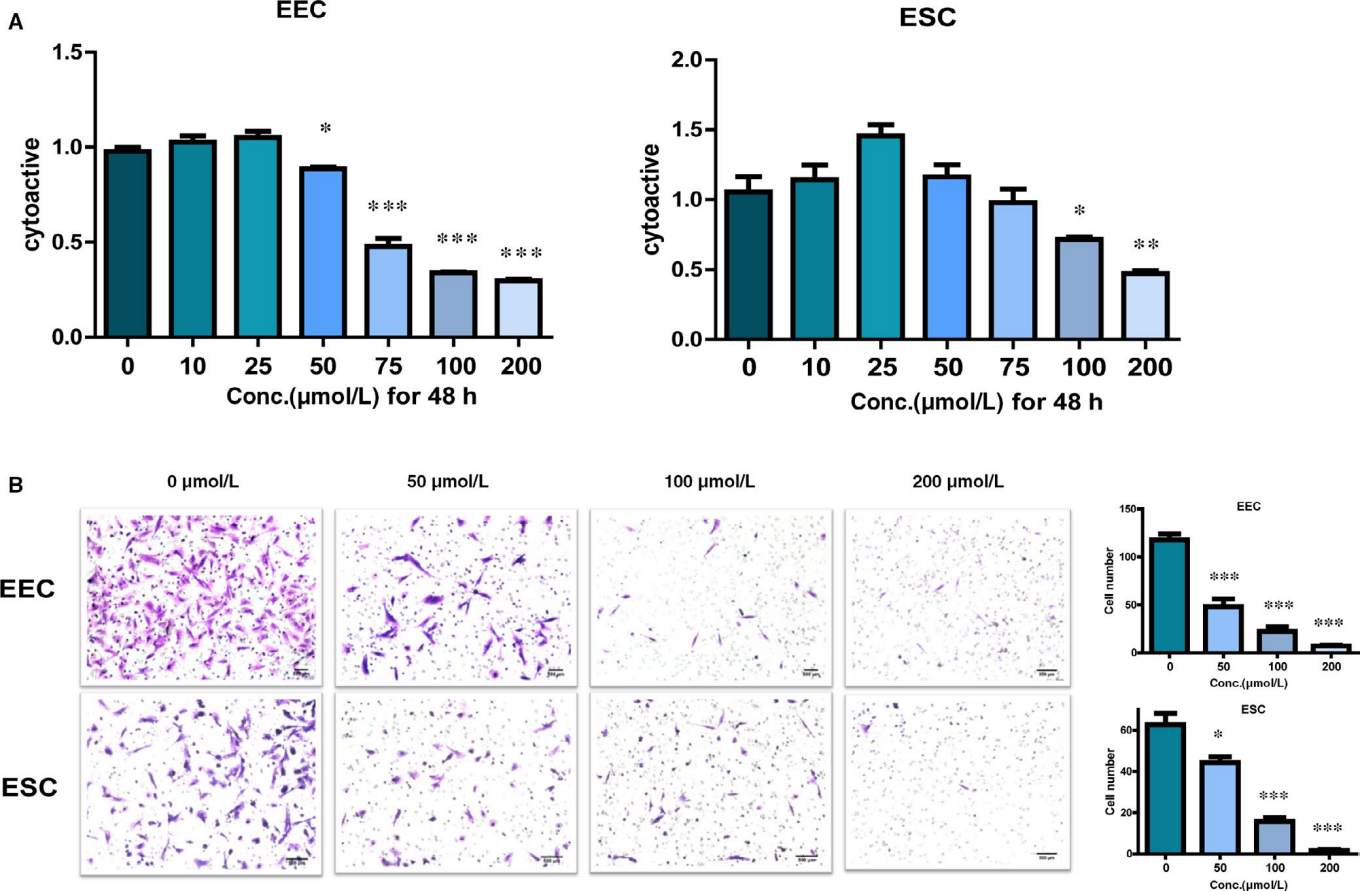
Mifepristone decreases the viability and migration of endometrial epithelial cells and stromal cells in adenomyosis. A, Cell viability was determined by CCK8 assay. Primary endometrial epithelial cells and stromal cells were treated with mifepristone at different concentrations (0, 10, 25, 50, 75, 100 and 200 μmol/L) for 48 h and subjected to CCK8 assay. The results showed that mifepristone inhibited the viability of endometrial epithelial cells and stromal cells in a dose‐dependent manner at 48 h. B, Left, phase‐contrast images of migrated endometrial epithelial cells and stromal cells treated with mifepristone at different concentrations (0, 50, 100 and 200 μmol/L) on the bottom membrane of Transwell insert (100× magnification). Right, the number of migrated endometrial epithelial cells and stromal cells in Transwell migration assay as indicated conditions. Number of migrated eutopic endometrial epithelial cells and stromal cells on the bottom membrane of Transwell insert was counted in 100× phase‐contrast images and 15 fields from each group (n = 3). Data were presented as the mean ± SEM. Conc., concentrations; EEC, endometrial epithelial cells; ESC, endometrial stromal cells. *P < .05, **P < .01, ***P < .001

To study the effect of mifepristone on the viability of endometrial epithelial cells and stromal cells of adenomyosis, CCK‐8 assay was performed. Human primary endometrial epithelial cells and stromal cells were treated with mifepristone in different concentrations (0, 10, 25, 50, 75, 100 and 200 μmol/L, respectively) for 48 hours. As shown in Figure 1A, the treatment of mifepristone exhibited a concentration‐dependent inhibitory effect on both endometrial epithelial and stromal cells compared with the controls. Especially, the viability of endometrial cells was significantly decreased when treated with mifepristone at concentrations above 50 μmol/L at 48 hours. These results indicated that mifepristone inhibits the viability of endometrial epithelial cells and stromal cells in adenomyosis.

### 3.2 Mifepristone inhibits the migratory capacity of eutopic endometrial epithelial cells and stromal cells in adenomyosis

Migration is a critical step during infiltration of eutopic endometrial epithelial cells and stromal cells into myometrium. To investigate the effects of mifepristone on the migratory capacity of eutopic endometrial epithelial cells and stromal cells in adenomyosis, migration assay was performed. Comparing with plain media controls, the number of migrated endometrial epithelial cells and stromal cells were both increased by FBS attraction in bottom wells. However, the migratory response of eutopic endometrial epithelial cells and stromal cells was significantly restricted after treatment with mifepristone in a dose‐dependent manner (Figure 1B). The results demonstrated that mifepristone inhibits the migration capacity of eutopic endometrial epithelial cells and stromal cells in adenomyosis.

### 3.3 Mifepristone down‐regulates the gene expressions of CDK1, CDK2, cyclin B, cyclin E and CXCR4 in endometrial epithelial cells by analysis of RNA‐Seq data

**Figure 2:**
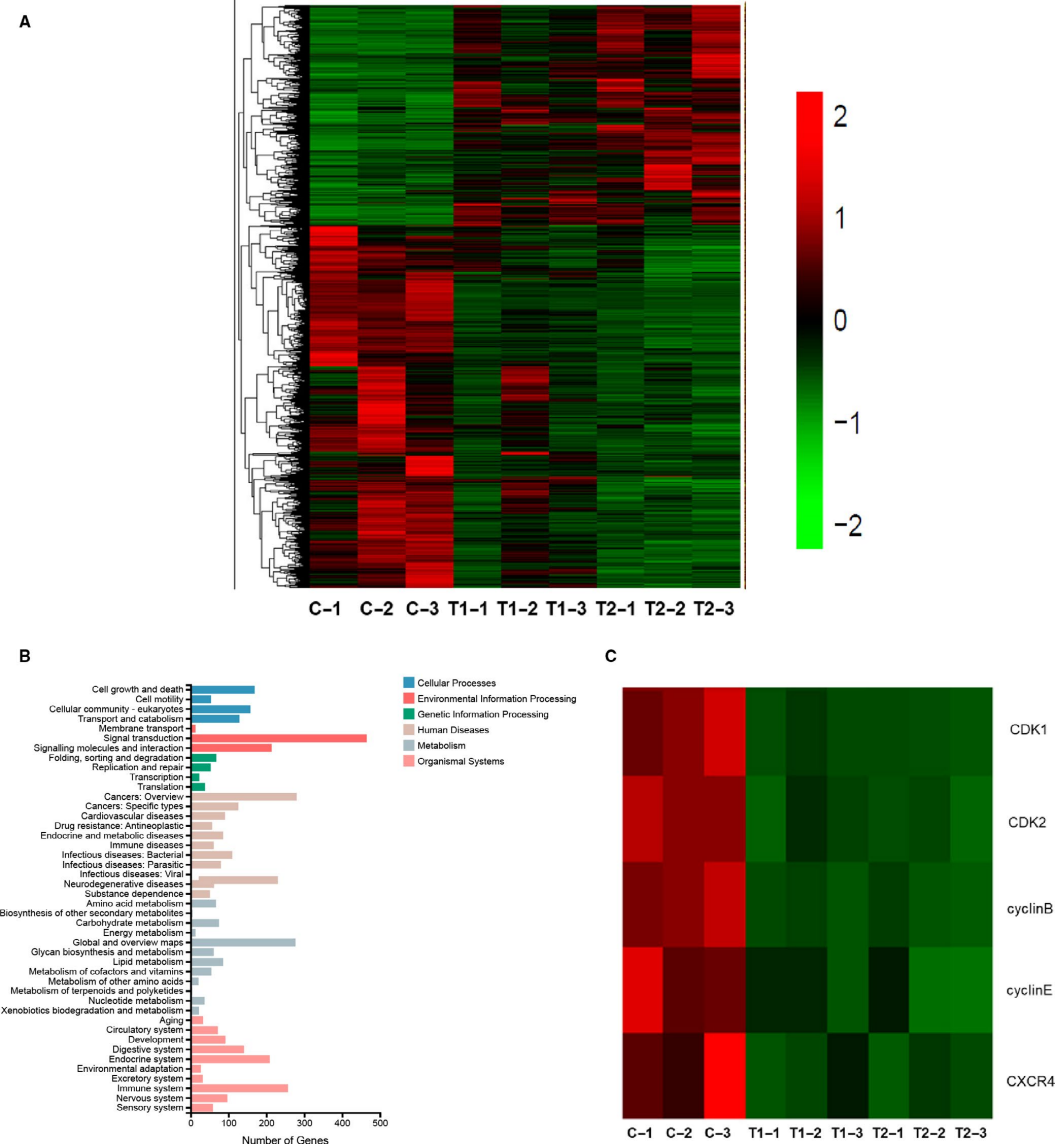
Mifepristone down‐regulates the expressions of CDK1, CDK2, cyclin B, cyclin E and CXCR4 in endometrial epithelial cells by analysis of RNA‐Seq data. A, Primary endometrial epithelial cells were treated with mifepristone (0, 50 and 100 μmol/L, respectively) for 24 h (n = 3). Red represents up‐regulation and green represents down‐regulation. B, KEGG analyse was conducted. C, The expressions of cyclin B, cyclin E, CDK1, CDK2 and CXCR4 were significantly down‐regulated in endometrial epithelial cells after treatment with mifepristone when compared to controls. C, Control (C‐1, the first patient in control group; C‐2, the second patient in control group; C‐3, the third patient in control group); T1, cells were treated with mifepristone at 50 μmol/L for 24 h (T1‐1, the first patient in T1 group; T1‐2, the second patient in T1 group; T1‐3, the third patient in T1 group); T2, cells were treated with mifepristone at 100 μmol/L for 24 h (T2‐1, the first patient in T2 group; T2‐2, the second patient in T2 group; T2‐3, the third patient in T2 group)

To investigate the potential mechanism of mifepristone treatment on the adenomyosis, gene expression was examined in the primary endometrial epithelial cells with or without treatment of mifepristone by RNA sequencing. The cells were treated with mifepristone (0, 50 and 100 μmol/L, respectively) for 24 hours (n = 3). Different genes between control group and mifepristone‐treated groups were clustered (Figure 2A). KEGG analyses found that the responses to mifepristone treatment were signal transduction, cell growth and death, cellular community and cell motility, etc (Figure 2B). Figure 2C showed that mifepristone prominently down‐regulated the expressions of CDK1, CDK2, cyclin B, cyclin E and CXCR4 in endometrial epithelial cells of adenomyosis when compared to controls, which are the key genes for regulating cell proliferation, apoptosis and migration.

### 3.4 Mifepristone induces the G1/G0 and G2/M phase arrest of cell cycle in endometrial epithelial cells and stromal cells of adenomyosis

**Figure 3:**
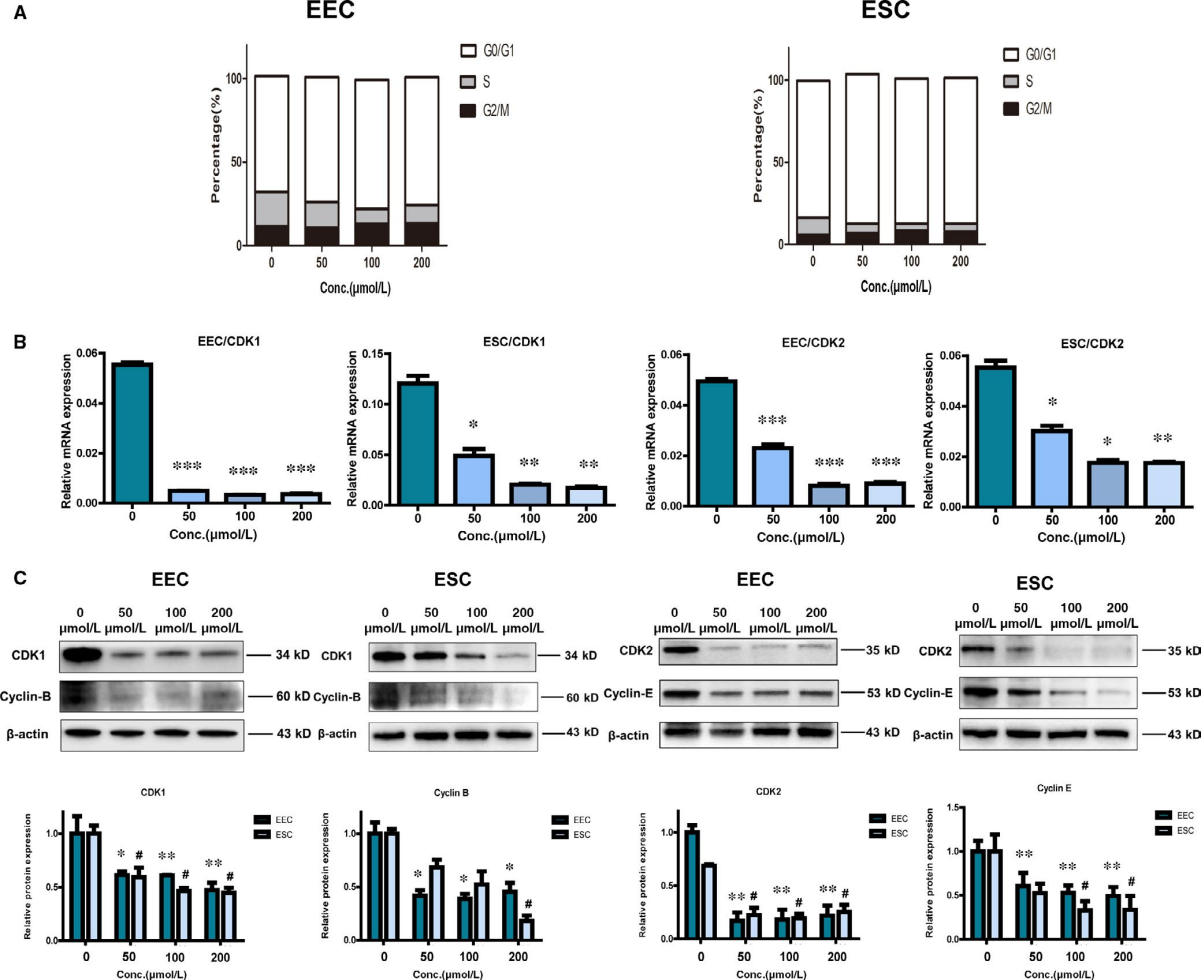
Mifepristone induces cell cycle arrest of endometrial epithelial and stromal cells through inhibiting CDK1 and CDK2 expressions. A, Cell cycle analysis was performed to quantify the cycle phase distribution of endometrial epithelial and stromal cells after mifepristone treatment. The cells were treated with mifepristone at different concentrations (0, 50, 100 and 200 μmol/L) and subjected to flow cytometric analysis. The results showed that mifepristone increased G2/M and G0/G1 phase cell population but decreased S phase cell population in a dose‐dependent manner. B, The down‐regulations of CDK1 and CDK2 were further confirmed by qRT‐PCR analysis in endometrial epithelial and stromal cells after treatment of mifepristone. C, Protein levels of CDK1, cyclin B and CDK2 and cyclin E were detected by Western blotting analysis in primary endometrial epithelial and stromal cells of adenomyosis after treatment of mifepristone. The band densitometry was quantified using Image J software and relative protein expression was graphed. Protein samples were from three different patients for each group. Data were presented as the mean ± SEM from three biologically independent samples. Conc., concentrations; EEC, endometrial epithelial cells; ESC, endometrial stromal cells. *P < .05, **P < .01, ***P < .001

Based on the above results, cell cycle analysis was performed to evaluate whether the anti‐proliferative effects of mifepristone on endometrial epithelial cells and stromal cells were caused by a specific phase arrest of cell cycle. Endometrial epithelial cells and stromal cells were treated with mifepristone at different concentrations (0, 50, 100 and 200 μmol/L) for 48 hours and subjected to flow cytometric analysis. The results showed that the proportions of cell cycle arrest in the G1/G0 and G2/M phase were increased in both endometrial epithelial cells and stromal cells after mifepristone treatment (Figure 3A). These data suggest that mifepristone mainly induces the G1/G0 and G2/M phase arrest of cell cycle in both endometrial epithelial cells and stromal cells of adenomyosis.

### 3.5 Mifepristone induces cell cycle arrest of endometrial epithelial cells and stromal cells through inhibiting CDK1 and CDK2 expressions

It is well known that the cell cycle is regulated by the action of a family of serine/threonine kinases known as cyclin‐dependent kinases (CDKs). According to the previous result of RNA sequencing (Figure 2C), the expressions of CDK1, CDK2, cyclin B and cyclin E were significantly decreased in endometrial epithelial cells treated with mifepristone when compared to control. The down‐regulations of CDK1 and CDK2 in the mifepristone‐treated group were further confirmed by qRT‐PCR analysis not only in primary endometrial epithelial cells but also in stromal cells (Figure 3B). Additionally, the results of Western blotting were also consistent with RNA sequencing and qRT‐PCR analysis (Figure 3C). Furthermore, this inhibitory effect of mifepristone was in a dose‐dependent response. Taken together, it is therefore speculated that mifepristone induces cell cycle arrest of endometrial epithelial cells and stromal cells through inhibiting CDK1 and CDK2 expressions.

### 3.6 Mifepristone induces apoptosis of both eutopic and ectopic endometrial cells in adenomyosis

**Figure 4:**
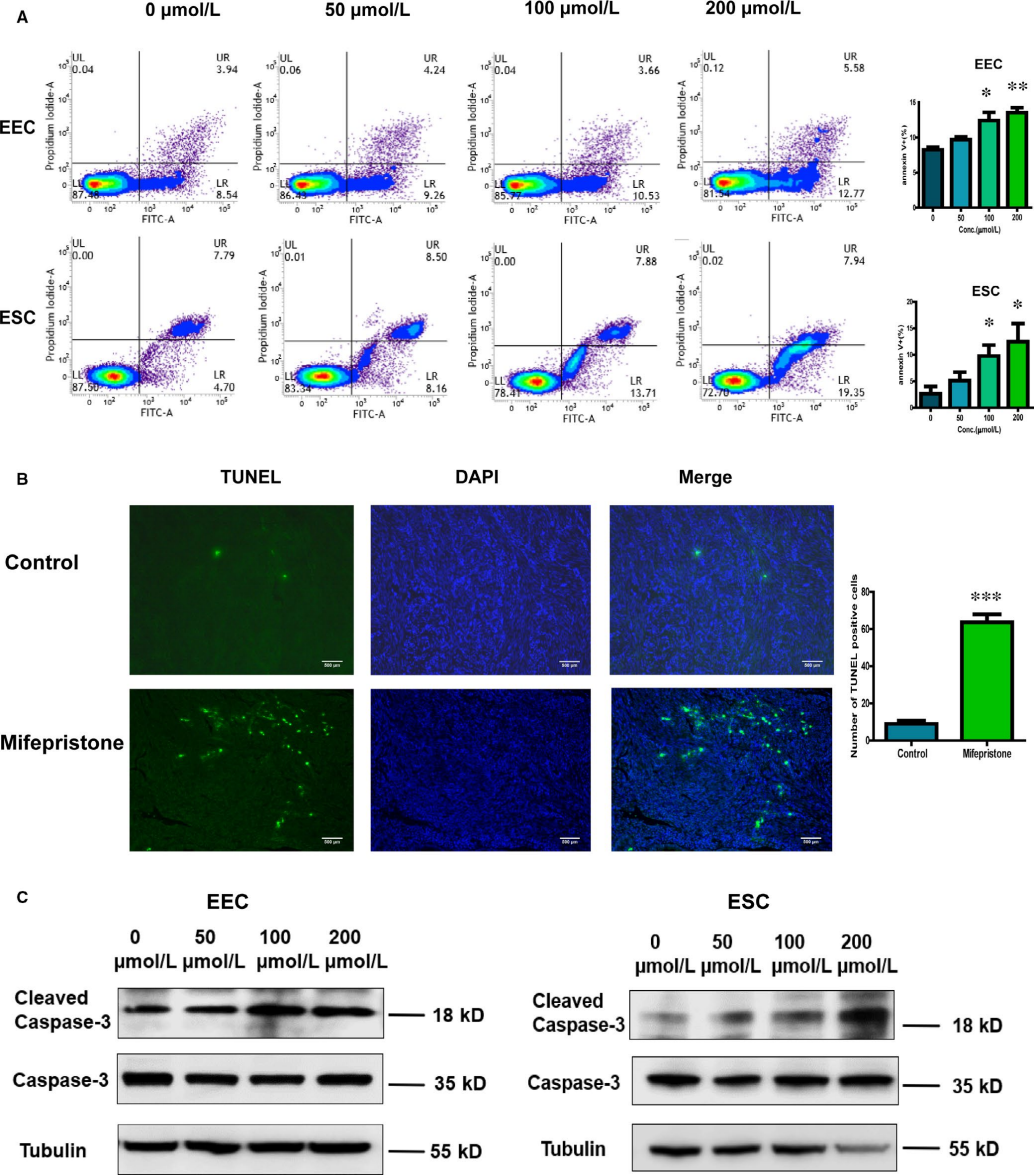
Mifepristone induces apoptosis of endometrial epithelial cells and stromal cells through the mitochondria‐dependent signalling pathway. A, The translocation of phosphatidylserine was determined by flow cytometric analysis of Annexin V binding and propidium iodide uptake in primary endometrial epithelial cells and stromal cells. The cells were treated with mifepristone at different concentrations (0, 50, 100 and 200 μmol/L) for 48 h. UL, viable cells, LR, early apoptotic cells, UR, terminal apoptosis cells. Representative images were shown and the rates of LR were calculated. All experiments were carried out three times independently. B, Representative micrographs of TUNEL staining for apoptosis in the ectopic lesions of myometrium in adenomyosis. TUNEL assay was performed to detect the level of cell apoptosis in the ectopic endometrium of adenomyosis patients treated with or without mifepristone. Green, apoptotic cells; blue, DAPI stained nuclei. C, Protein levels of apoptosis‐related proteins (caspase‐3 and cleaved caspase 3) were detected by Western blotting analysis in primary endometrial epithelial cells and stromal cells. Bar = 500 μm. Data were shown as mean ± SEM. EEC, endometrial epithelial cells; ESC, endometrial stromal cells. *P < .05, **P < .01, ***P < .001

Cell apoptosis is in close association with cell viability. To investigate the effect of mifepristone on the induction of apoptosis in eutopic endometrial epithelial cells and stromal cells of adenomyosis, flow cytometric analysis was examined. We observed from Figure 4A that late apoptotic and necrotic cells remained unchanged at all concentrations of mifepristone tested, while the proportion of early apoptotic cells increased with increasing drug concentrations when compared with controls. The results revealed that mifepristone induces apoptotic cell death of eutopic endometrial epithelial cells and stromal cells in adenomyosis.

To further evaluate the effect of mifepristone on uterus lesions of adenomyosis patients, TUNEL assay was performed to detect the level of cell apoptosis in ectopic endometrial cells of patients treated with or without mifepristone. TUNEL‐positive cells were visualized as indicated by green fluorescence staining (Figure 4B), and the number of TUNEL‐positive cells was determined. The results indicated that apoptotic cells were significantly increased in ectopic endometrium of mifepristone‐treated adenomyosis group when compared to mifepristone‐untreated group (*P* < .000). The findings suggested that mifepristone not only causes apoptosis of eutopic endometrial cells in vitro but also induces apoptosis of ectopic endometrial cells in adenomyosis.

### 3.7 Mifepristone induces apoptosis of endometrial epithelial cells and stromal cells through the mitochondria‐dependent signalling pathway

To further validate the mechanism of apoptosis induced by mifepristone in endometrial cells of adenomyosis, Western blotting was performed to detect the effect of mifepristone on the expression of a molecular marker of cell apoptosis. As the drug concentration increased, the expression of caspase‐3 protein was mildly decreased, while cleaved caspase‐3 was significantly increased in both primary endometrial epithelial cells and stromal cells of adenomyosis (Figure 4C). Since caspase‐3 is a critical mediator of mitochondrial events of cell apoptosis, the data indicated mifepristone induces apoptosis of endometrial epithelial cells and stromal cells through the mitochondria‐dependent signalling pathway.

### 3.8 Mifepristone inhibits the migratory capacity of the eutopic endometrial epithelial cells and stromal cells through suppressing CXCR4 expression in adenomyosis

**Figure 5:**
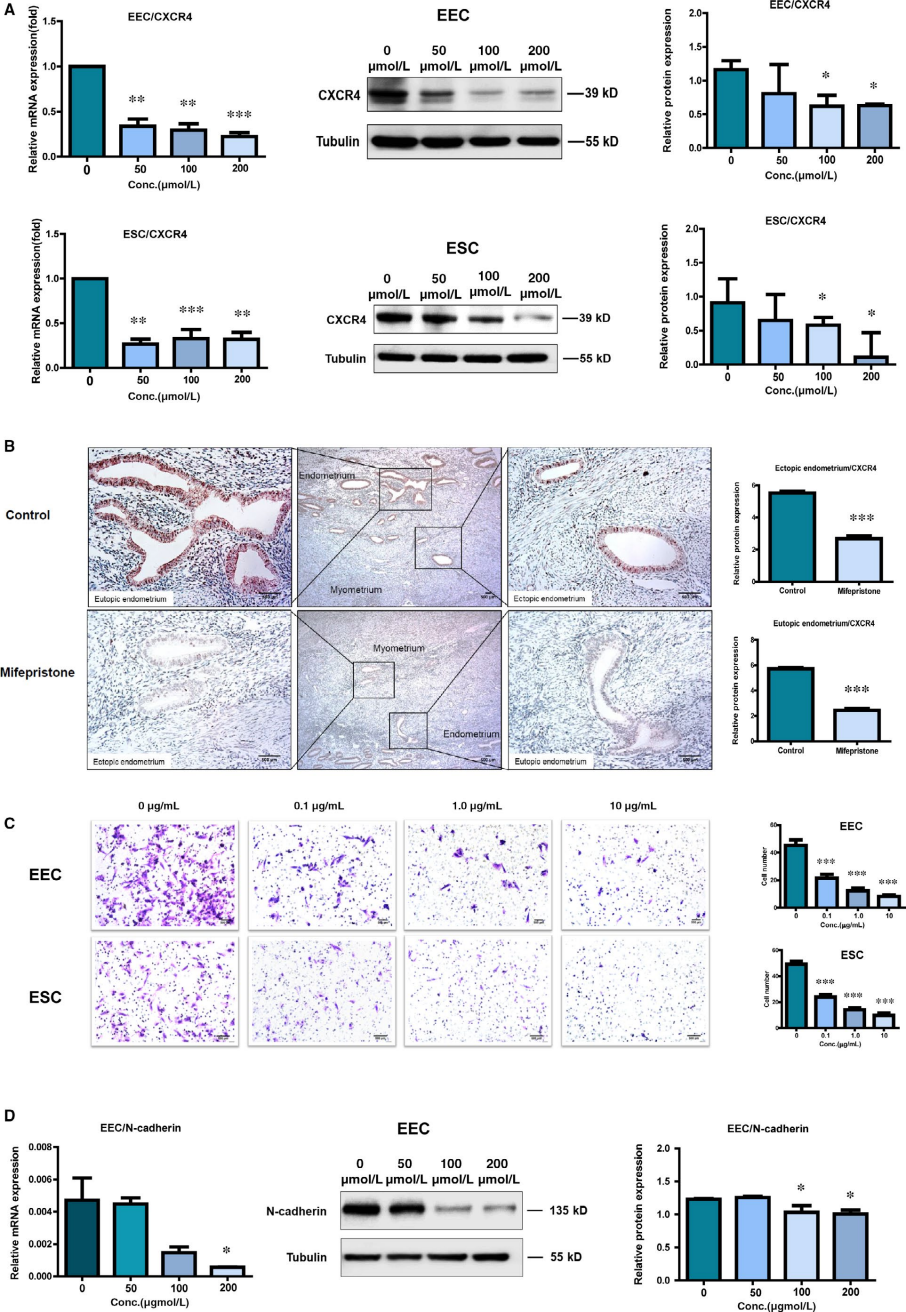
Mifepristone restricts the migration capacity of endometrial epithelial cells and stromal cells through suppressing CXCR4 expression and also inhibits EMT process of endometrial epithelial cells in adenomyosis. A, mRNA and protein expressions of CXCR4 were detected by qRT‐PCR and Western blotting respectively in endometrial epithelial cells and stromal cells with or without mifepristone treatment at different concentrations (0, 50, 100 and 200 μmol/L). B, Protein expression of CXCR4 was examined in the section of eutopic and ectopic endometriums in adenomyosis patients with or without treatment of mifepristone by immunohistochemistry. Image was captured at 200× magnification. C, Left, phase‐contrast images of migrated endometrial epithelial cells and stromal cells treated with AMD3100 at different concentrations (0, 0.1, 1.0 and 10 μg/mL) on the bottom membrane of Transwell insert (100× magnification). Right, the number of migrated endometrial epithelial cells and stromal cells in Transwell migration assay as indicated conditions. Number of migrated endometrial epithelial cells and stromal cells on the bottom membrane of Transwell insert was counted in 100× phase‐contrast images and 15 fields from each group (n = 3). D, mRNA expression of N‐cadherin was detected by qRT‐PCR and the protein expression was checked by Western blotting in primary endometrial epithelial cells of adenomyosis with or without mifepristone treatment. The data shown represent mean ± SEM from three samples. EEC, endometrial epithelial cells; ESC, endometrial stromal cells. *P < .05, **P < .01, ***P < .001

The result of RNA sequencing showed that mRNA expression of CXCR4 was down‐regulated in the endometrial epithelial cells of adenomyosis by mifepristone treatment. The down‐regulations of CXCR4 in the mifepristone‐treated endometrial epithelial cells and stromal cells were confirmed by qRT‐PCR analysis (Figure 5A). Protein expression of CXCR4 was also examined by Western blotting, and the results showed that CXCR4 expression was decreased in the eutopic endometrial epithelial cells and stromal cells in a dose‐dependent manner when treated with mifepristone (Figure 5A). Subsequently, immunohistochemistry was conducted to detect the protein expression of CXCR4 in eutopic and ectopic endometriums of adenomyosis patients with and without mifepristone treatment. Semi‐quantitative detection showed that mifepristone‐treated adenomyosis group significantly decreased the expression of CXCR4 in both eutopic and ectopic endometriums when compared to mifepristone‐untreated group (Figure 5B).

CXCR4, one of the most prevalent chemokine receptors, can promote the migration of many different types of cancer cells while it was significantly down‐regulated in the primary endometrial epithelial cells of adenomyosis after treated with mifepristone by analysis of RNA‐Seq data (Figure 2C). To confirm the role of CXCR4 in the migration of adenomyosis, we detected the migration ability of eutopic endometrial epithelial cells and stromal cells treated with different doses of CXCR4 inhibitor (AMD3100) using transwell assay. As shown in Figure 5C, we found that AMD3100 greatly suppressed the migratory activity of eutopic endometrial epithelial cells and stromal cells in a dose‐dependent manner, which suggested that CXCR4 promotes the migratory capacity of eutopic endometrial epithelial cells and stromal cells in adenomyosis. Since both mifepristone and AMD3100 inhibit the migration ability of endometrial epithelial cells and stromal cells in adenomyosis and CXCR4 expression was down‐regulated by mifepristone treatment, it is evident that mifepristone inhibits the migratory capacity of the eutopic endometrial epithelial cells and stromal cells through suppressing CXCR4 expression in adenomyosis.

### 3.9 Mifepristone inhibits epithelial‐mesenchymal transition of endometrial epithelial cells in adenomyosis

Epithelial‐mesenchymal transition is a vital step in the invasion and metastasis of endometrial epithelial cells in adenomyosis. In an attempt to investigate the effect of mifepristone on EMT process of eutopic endometrial epithelial cells in adenomyosis, EMT‐related markers were checked. The mRNA level of N‐cadherin was significantly down‐regulated in endometrial epithelial cells when treated with mifepristone in a dose‐dependent manner. Similar trend was also found in N‐cadherin protein expression detected by Western blotting (Figure 5D). However, there was no increase in the protein level of E‐cadherin in endometrial epithelial cells treated with mifepristone when compared with untreated controls (data were not shown). The findings signified that mifepristone inhibits the EMT process of endometrial epithelial cells in adenomyosis.

### 3.10 Mifepristone serves as a novel therapeutic drug in the treatment of adenomyosis

**Figure 6:**
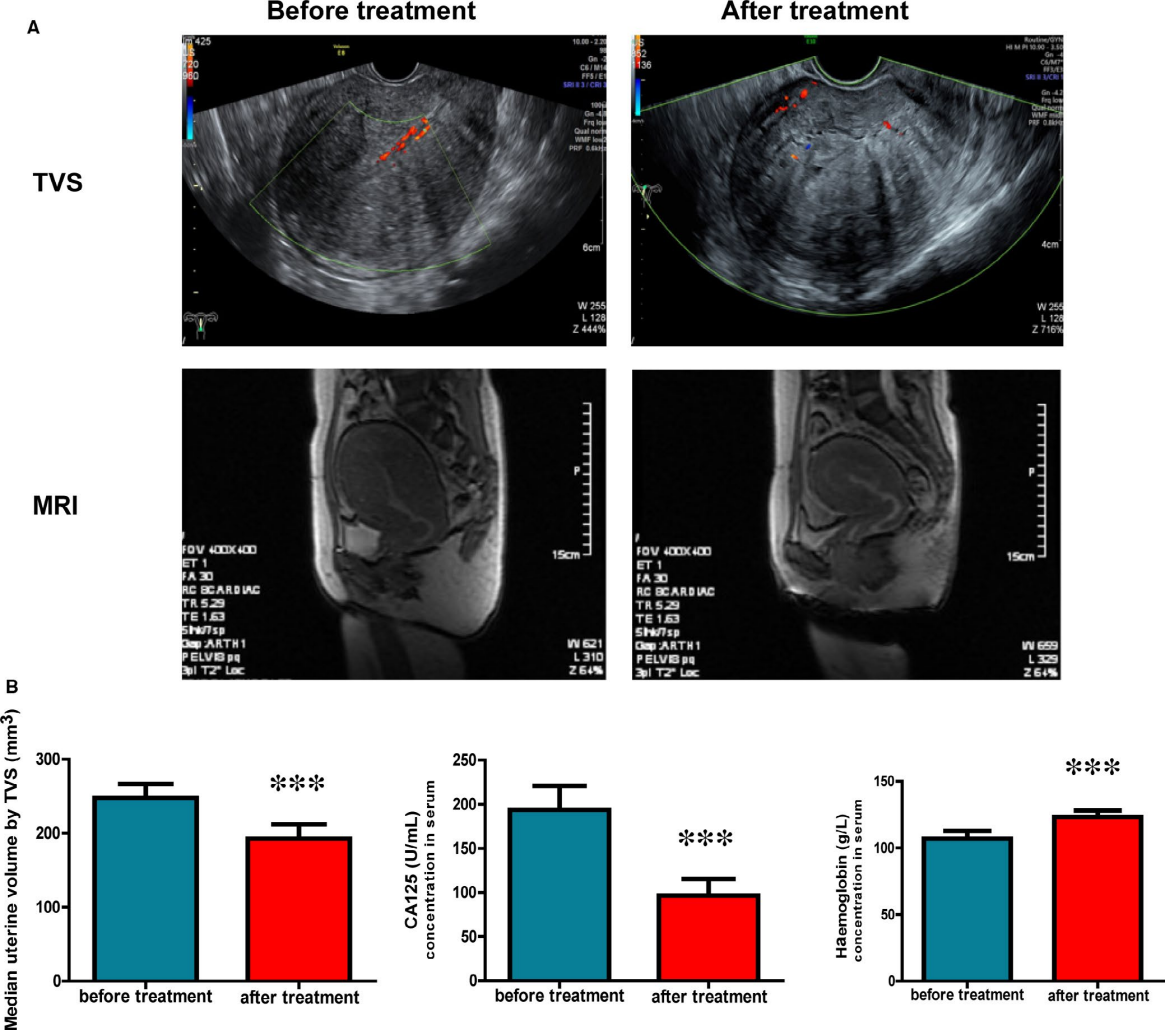
Mifepristone could be applied in the treatment of adenomyosis. A, Representative uterine images of TVS and MRI in the same adenomyosis patient before and after three‐month treatment with mifepristone. B, Median uterine volume was calculated in 20 adenomyosis patients before and after mifepristone treatment. Ultrasonography was applied to measuring the length of the long‐ and short‐axis of the sagittal section and the long‐axis of the cross section of the uterus. Uterine volume was assessed using the formula for an ovoid (length × width × depth × 0.52). The CA125 concentration and haemoglobin concentration in serum of adenomyosis patient were measured before and after three‐month treatment with mifepristone. MRI, magnetic resonance imaging; TVS, transvaginal ultrasound. Data were expressed as mean ± SEM. *P < .05, **P < .01 and ***P < .001

It is known that uterine volume is always increased and associated with severity of adenomyosis. To further study the therapeutic efficacy of mifepristone on adenomyosis patients, uterine volume was measured by transvaginal ultrasound (TVS) and MRI in this study. As indicated in the representative uterine images of TVS and MRI (Figure 6A), the uterine volume was decreased after three‐month treatment with mifepristone in the same adenomyosis patient. As shown in Figure 6B, the mean uterine volume before treatment was 247.7 ± 18.9 mm^3^ while the mean volume was 192.7 ± 19.3 mm^3^ after three‐month treatment based on the TVS measurement, and there is a statistically significant reduction (*P* < .001). Furthermore, the CA125 and haemoglobin concentrations in serum were two important markers for the development of adenomyosis. The mean CA125 concentration in serum before treatment was 193.8 ± 27.2 U/mL while the mean concentration was 96.6 ± 18.7 U/mL after three‐month treatment, and haemoglobin concentration in serum was increased from 106.9 ± 6.0 to 123.4 ± 4.7 g/L. The results showed that CA125 concentration in serum was dramatically decreased while haemoglobin concentration was significantly increased after mifepristone treatment (Figure 6B). Our data showed that mifepristone decreases the uterine volume and CA125 concentration while increases haemoglobin concentration in serum for adenomyosis patient and therefore could serve as a novel therapeutic drug in the treatment of adenomyosis.

## 4 DISCUSSION

Adenomyosis is benign invasion of the endometrium into the myometrium, and it shows malignant features, such as abnormal growth, invasion and migration.^25^, ^26^ Increased proliferative and invasive characteristics of the eutopic endometrial cells are likely key pathogenic factors for adenomyosis.^27^, ^28^ These changes enhance the infiltration of endometrial epithelial cells and stromal cells into myometrium. Finally, sustained proliferation and continued migration of endometrial cells and compensatory hypertrophy or hyperplasia of peripheral muscular cells result in increased numbers of foci and produce a diffusely or locally enlarged uterus of adenomyosis patient, which is one of the most clinical manifestations of adenomyosis.^29^, ^30^

Current medical treatments for adenomyosis often fail to fully resolve symptoms, result in unacceptable side‐effects and represent a large healthcare burden.^31^, ^32^ Mifepristone, an old drug, is effective, well tolerated and affordable. Recent researches demonstrate that mifepristone has more benefits for human health than what we originally thought following current understanding of mechanism of mifepristone action. It is reported that the mechanisms of effectiveness are involved in anti‐progesterone, down‐regulation of CDK2, Bcl‐2, growth inhibition of various cancer cell lines, suppression of invasive and metastatic cancer cells, etc.^33^ However, there are few studies about the application of mifepristone in adenomyosis patients and its mechanism of therapeutic efficacy. Firstly, we found that mifepristone directly inhibited the viability and migration of the endometrial epithelial cells and stromal cells in adenomyosis and the effects of mifepristone were concentration‐dependent. To further make clear how mifepristone plays the roles, RNA sequencing was performed and we found that mifepristone treatment regulated proliferation‐related and migration‐correlated genes, and further experiments were conducted to unveil the underlying mechanism.

Cell viability is related with proliferation and apoptosis. The effective concentration of mifepristone in inhibiting the viability of human endometrial cells in this study is similar to that used in treatments of kinds of cancers.^15^, ^34^, ^35^ We further observed that mifepristone mainly induced the G0/G1 and G2/M phase arrest of the cell cycle of adenomyotic endometrial epithelial cells and stromal cells in vitro. Our results demonstrated that mifepristone induces cell cycle arrest of endometrial epithelial cells and stromal cells through inhibiting CDK1 and CDK2 expression. Moreover, we found that mifepristone induced early apoptotic cell death in eutopic endometrial epithelial cells and stromal cells of adenomyosis in a dose‐dependent manner by flow cytometry. TUNEL assay confirmed that mifepristone could induce apoptosis of ectopic endometrial cells in adenomyosis as well. The present study also demonstrated that mifepristone significantly increased cleaved caspase‐3 in both primary endometrial epithelial cells and stromal cells of adenomyosis in a dose‐dependent manner, which is the critical mediator of mitochondrial events of cell apoptosis. Therefore, our study indicated mifepristone induces apoptosis of endometrial epithelial cells and stromal cells through the mitochondria‐dependent signalling pathway.

Migration of endometrial cells is another vital feature in the cause and development of adenomyosis.^28^ In the present study, we found mifepristone down‐regulated the expressions of CXCR4 in endometrial epithelial cells by analysis of RNA‐Seq data. qRT‐PCR analysis and Western blotting further validated that mifepristone could inhibit CXCR4 expression in primary endometrial epithelial cells and stromal cells of adenomyosis in a dose‐dependent manner. Immunohistochemistry staining also showed that CXCR4 expression was significantly decreased in mifepristone‐treated adenomyosis group. In addition, transwell assay was then performed to confirm that CXCR4 increased the migration of eutopic endometrial epithelial cells and stromal cells in adenomyosis. Thus, inhibiting CXCR4 expression could prevent the migration of ectopic endometrial cells into the myometrium. Furthermore, mifepristone inhibited the migration of endometrial epithelial cells and stromal cells. Therefore, we conclude that mifepristone inhibits the migration capacity of the eutopic endometrial epithelial cells and stromal cells through suppressing CXCR4 expression in adenomyosis.

Epithelial‐mesenchymal transition serves as an important role in the development of adenomyosis by promoting cell invasion.^36^, ^37^ It is reported that E‐cadherin‐negative and N‐cadherin‐positive endometriotic epithelial cells were greatly invasive in vitro.^38^ Previous studies found that mifepristone blocks the EMT‐related signalling pathways to inhibit the invasion of peritoneal mesothelial cells and endometrial cancer cells.^39^ In our study, the results indicated that N‐cadherin, a characteristic molecular feature of EMT, was significantly decreased in endometrial epithelial cells treated with mifepristone in a dose‐dependent manner. Therefore, mifepristone restricts the invasion of endometrial epithelial cells via suppression of EMT process in adenomyosis.

It is known that large uterine is a feature for the adenomyosis and results from sustained proliferation and continued migration of endometrial cells. Uterine volume is always increased and associated with severity of adenomyosis. Furthermore, CA125 is a traditional marker for the diagnosis and progression of adenomyosis. In this study, we demonstrated that uterine volume and CA125 concentration in serum were significantly decreased after treatment of mifepristone for adenomyosis patients. In addition, hypermenorrhoea always happens and results in anaemia in adenomyosis patients. The results showed that haemoglobin concentration was significantly increased after mifepristone treatment. Therefore, our data proved that mifepristone might serve as a novel therapeutic drug in the treatment of adenomyosis.

**Figure 7:**
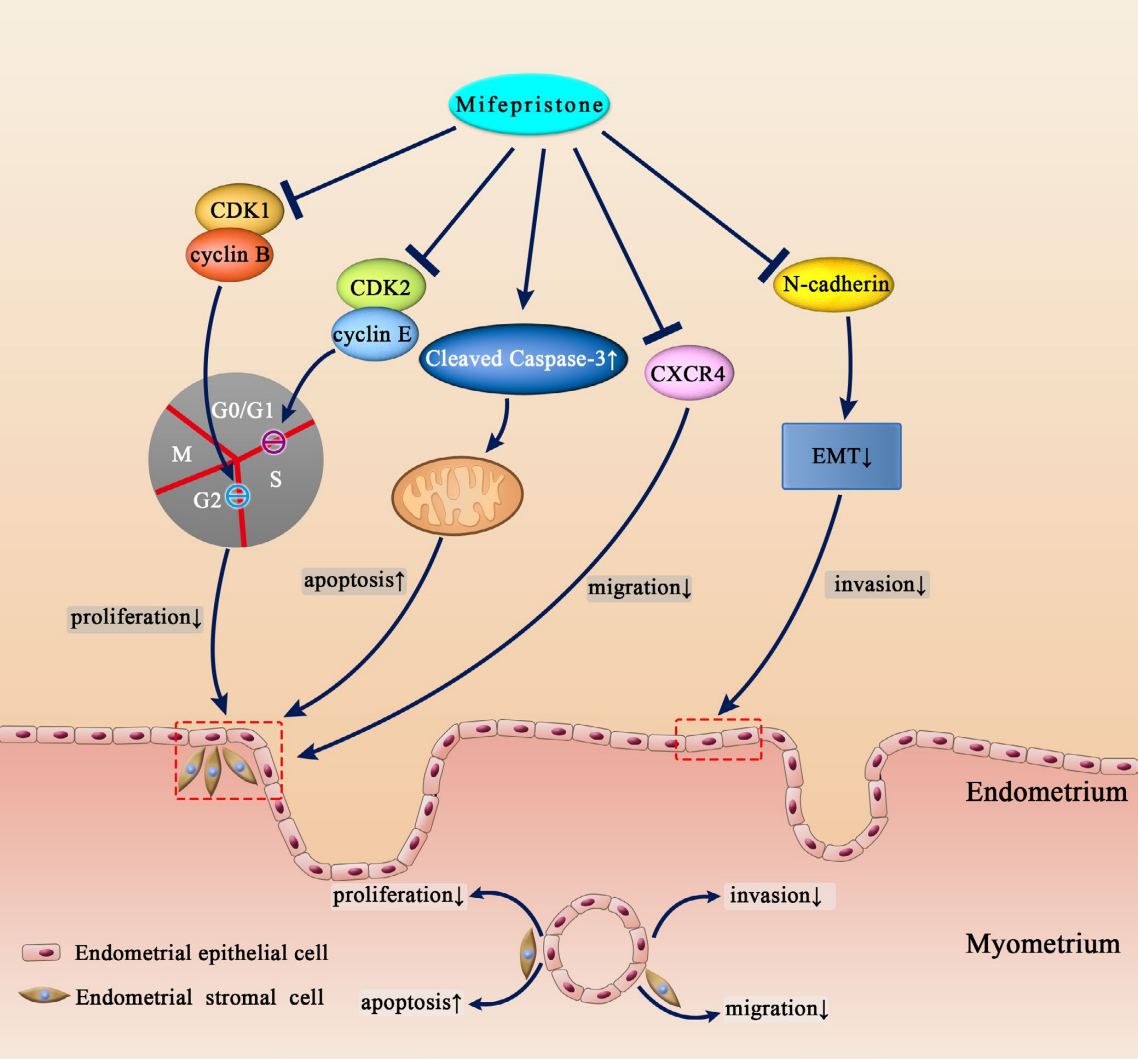
Proposed model of mifepristone for the treatment of adenomyosis. Mifepristone causes cell cycle arrest through inhibiting CDK1 and CDK2 expression and induces cell apoptosis via the mitochondria‐dependent signalling pathway in endometrial epithelial cells and stromal cells of adenomyosis. Furthermore, mifepristone inhibits the migration of endometrial epithelial cells and stromal cells through decreasing CXCR4 expression and restricts the invasion of endometrial epithelial cells via suppression of EMT process in adenomyosis

In summary, the present study first investigated the direct effects of mifepristone on primary endometrial epithelial cells and stromal cells in adenomyosis. Mifepristone causes cell cycle arrest through inhibiting CDK1 and CDK2 expression and induces cell apoptosis via the mitochondria‐dependent signalling pathway in endometrial epithelial cells and stromal cells of adenomyosis. Furthermore, mifepristone inhibits the migration of endometrial epithelial cells and stromal cells through decreasing CXCR4 expression and restricts the invasion of endometrial epithelial cells via suppression of EMT process in adenomyosis (Figure 7). We also report that mifepristone treatment decreases the uterine volume, CA125 concentration and increases haemoglobin concentration in serum for adenomyosis patient. Therefore, we demonstrate that mifepristone could serve as a novel therapeutic drug in the treatment of adenomyosis and the old dog can do a new trick.

## CONFLICT OF INTEREST

The authors declare no potential conflicts of interest with respect to the research, authorship and/or publication of this article.

## AUTHOR CONTRIBUTIONS

Xuan Che, Jianzhang Wang and Jiayi He performed experiments. Qin Yu, Wenting Sun and Gen Zou collected the clinical samples. Xuan Che and Shuyi Chen analysed the data. Xuan Che, Jianzhang Wang, Xinyue Guo and Tiantian Li made the figures and drafted the article. Xinmei Zhang, Jianzhang Wang and Xuan Che conceived and critically reviewed the project. All authors have read and approved the final version of the paper.

## Data Availability

The data that support the findings of this study are available from the corresponding author upon reasonable request.
